# Modifiable behavioral risk factors for NCDs and sleep in Brazilian adolescents

**DOI:** 10.11606/s1518-8787.2023057004957

**Published:** 2023-09-14

**Authors:** Raina Jansen Cutrim Propp Lima, Mônica Araujo Batalha, Cecília Cláudia Costa Ribeiro, Pedro Martins Lima, Antônio Augusto Moura da Silva, Rosângela Fernandes Lucena Batista

**Affiliations:** I Instituto Federal de Educação. Ciência e Tecnologia do Maranhão Departamento de Ensino Açailândia MA Brasil Instituto Federal de Educação. Ciência e Tecnologia do Maranhão. Departamento de Ensino. Açailândia, MA, Brasil; II Universidade Federal do Maranhão Centro de Ciências Biológicas e da Saúde Departamento de Saúde Pública São Luís MA Brasil Universidade Federal do Maranhão. Centro de Ciências Biológicas e da Saúde. Departamento de Saúde Pública. São Luís, MA, Brasil; III Universidade Federal do Maranhão Centro de Ciências Biológicas e da Saúde Departamento de Odontologia II São Luís MA Brasil Universidade Federal do Maranhão. Centro de Ciências Biológicas e da Saúde. Departamento de Odontologia II. São Luís, MA, Brasil; IV Universidade Federal do Maranhão Centro de Ciências Sociais, Saúde e Tecnologia Imperatriz MA Brasil Universidade Federal do Maranhão. Centro de Ciências Sociais, Saúde e Tecnologia. Imperatriz, MA, Brasil

**Keywords:** Sleep, Adolescent Health, Sleepiness, Sedentary Behavior, Alcohol Consumption, Sugar-Sweetened Beverages

## Abstract

**OBJECTIVE:**

To analyze the association between modifiable behavioral risk factors for non-communicable diseases and sleep parameters in Brazilian adolescents.

**METHODS:**

This was a cross-sectional study that used data from the RPS Cohort Consortium, São Luís, Brazil for the follow-up of adolescents aged 18–19 years (n = 2,515). The outcomes were excessive daytime sleepiness (Epworth Sleepiness Scale – ESS) and sleep quality (Pittsburgh Sleep Quality Index – PSQI). The exposures of interest were the behavioral risk factors for non-communicable diseases (NCDs): screen time, physical inactivity, alcohol, smoking, illicit drugs, caffeine intake, and consumption of sugar-sweetened beverages. Excess weight was considered a possible mediator of this association between the exposures of interest and the outcomes. The models were analyzed by modeling with structural equations.

**RESULTS:**

Physical inactivity (standardized coefficient, SC = 0.112; p = 0.001), higher consumption of alcohol (SC = 0.168; p = 0.019) and of sugar-sweetened beverages (SC = 0.128; p < 0.001) were associated with excessive daytime sleepiness in adolescents; better socioeconomic status was also associated with this outcome (SC = 0.128; p < 0.001). Physical inactivity (SC = 0.147; p < 0.001) and higher consumption of sugar-sweetened beverages (SC = 0.089; p = 0.003) were also associated with poor sleep quality. Overweight was neither a mediator nor associated with sleep quality or excessive daytime sleepiness.

**CONCLUSIONS:**

The main modifiable behavioral risk factors for NCDs are associated with worse sleep parameters already in adolescence, which serves as a warning toward the accumulation of risks for sleep disorders in the future.

## INTRODUCTION

Sleep is an essential biological process for survival and is important for the physical, mental, and social well-being of individuals^1^. Since it is essential for healthy development, the American Academy of Sleep Medicine recommends that adolescents should regularly sleep 8 to 10 hours a day, an amount associated with better health outcomes and quality of life^2^.

Adolescence is a period marked by changes in sleep patterns due to biological, environmental, and psychosocial factors, such as pubertal maturation, circadian rhythm regulation, and less regular schedules^3^. These changes are characterized by diurnal sleep dysfunctions, which, in turn, are risk factors for excessive daytime sleepiness in this age group^4^. Adolescents are not getting enough sleep, and this is a chronic problem worldwide. However, many of the factors that contribute to the current “epidemic” of insufficient sleep in adolescents are modifiable^5^.

The adolescent’s behavioral, psychosocial, and metabolic factors, such as the use of licit and illicit drugs, overweight, high intake of caffeine^3^ and added sugar^6^, sedentary behavior^7^, screen time^8^, and socioeconomic status^9^ have been associated with poor sleep quality patterns and excessive daytime sleepiness.

Loss of sleep and its disorders can have their effects accumulated over time, being associated with several harmful health consequences, such as non-communicable diseases (NCDs)^10^. Some of the factors that lead to changes in sleep are considered as risks for major NCDs. According to the World Health Organization (WHO)^11^, NCDs are causally associated with four modifiable behavioral risk factors: harmful use of alcohol, smoking, unhealthy diet, and physical inactivity, which can trigger metabolic changes such as obesity.

The obesity epidemic is a reality in all age groups and has been considered one of the main causes of the NCDs numbers in the world^12^. Poor quality sleep has been linked to higher rates of being overweight, just as obesity can trigger comorbidities that affect sleep quality. In this bidirectional relationship^13^, behavioral risk factors plausibly influence both obesity and sleep; such factors will be investigated in this study.

The most frequent behavioral health risks in adolescence act together as part of an unhealthy style and increase the risk of obesity. Therefore, better understanding the complex association between these risk factors and sleep in an adolescent population becomes necessary, since this combination can impact health throughout life. In addition, no study to our knowledge of has used overweight as a mediator of these associations.

This study, therefore, aims to estimate, by structural equation modeling, the association between modifiable behavioral risk factors for NCDs and sleep quality and excessive daytime sleepiness in adolescents, considering overweight as a mediator of these pathways.

## METHODS

### Study design

Cross-sectional study nested in a cohort study developed in the municipality of São Luís, state of Maranhão. This cohort is part of the research entitled “*Determinantes ao longo do ciclo vital da obesidade, precursores de doenças crônicas, capital humano e saúde mental*” (“Determinants throughout the life cycle of obesity, precursors of chronic diseases, human capital, and mental health,”developed by the RPS Consortium (Ribeirão Preto, Pelotas, and São Luís) of Brazilian Birth Cohorts, which comprises the Ribeirão Preto School of Medicine of the Universidade de São Paulo (USP), the Universidade Federal de Pelotas (UFPel), and the Universidade Federal do Maranhão (UFMA).

In São Luís, the birth cohort began in 1997 (n = 2,443) and includes two follow-ups: the first at 7–9 years and the second at 18–19 years of age^14^.

In this study, we used data collected in the second follow-up of the cohort, obtained in 2016. All participants in the first phase of the study were sought in the four Military Enlistment Boards on the island of São Luís, in the 2014 school census, and in universities. Those identified were invited to attend the follow-up, totaling 687 participants.

To increase the power of the sample and to prevent future losses, the cohort was opened to include other individuals born in 1997. In a first stage, these individuals were included by lottery, by using the database of the *Sistema de Informações sobre Nascidos Vivos* (SINASC – Live Birth Information System). In a second stage, volunteers identified in schools and universities were included, totaling 1,828 participants. The final sample consisted of 2,515 adolescents.

Data were collected by health students and professionals duly trained, on the premises of UFMA, who conducted interviews to apply structured questionnaires answered by the participants themselves. The following information was used: schooling of the head of the family and the adolescent, monthly family income, economic class, alcohol consumption, smoking, use of illicit drugs, time of exposure to screens, food consumption, level of physical activity, quality of sleep, and daytime sleepiness.

### Variables Used

#### Socioeconomic status

The socioeconomic situation (SES) construct is a latent variable, derived from the variables observed: level of education of the head of the family and the adolescent (elementary, secondary, and higher education); monthly family income in minimum wages – in 2016, it corresponded to R$ 880.00 (< 1; 1 to 2.9; 3 to 4.9; ≥ 5); and economic class of the family, categorized according to the Brazilian Economic Classification Criterion (CCEB) 2016^15^, in D/E (poorer and less educated class) or C, B, and A (richer and more educated class).

#### Alcohol consumption

The pattern of alcohol consumption was assessed by using the Alcohol Use Disorder Identification Test (AUDIT) and categorized as low risk (0–7) and high risk (8–40)^16^.

#### Smoking

Evaluated by investigating smoking at the time of the study, regardless of frequency and/or quantity, and dichotomized into yes or no.

#### Use of illicit drugs

Use of illicit drugs (marijuana, cocaine, heroin, ecstasy, crack, LSD) was assessed by a self-administered questionnaire and dichotomized into “never used” and “has used or currently uses.”

#### Consumption of sugar-sweetened beverages

The frequency of daily consumption of sugar-sweetened beverages and caffeine were obtained with a food frequency questionnaire validated for adolescents^17^ and adapted to the food consumption of adolescents from São Luís, Maranhão. The questionnaire contained 106 food items, which assess the frequency and portion of consumption of these foods in the last 12 months. The consumption of sugar-sweetened beverages was estimated by the intake of soft drinks, industrialized juices, chocolates, and energy drinks. The percentage of energy from these beverages in relation to the daily energy intake of the adolescent was calculated by the sum of energy from all sugar-sweetened beverages, multiplied by 100 and divided by the sum of total daily energy intake. The result was categorized into < 5%, ≥ 5% and < 10%, and ≥ 10%^18^.

#### Caffeine consumption

To estimate caffeine intake in milligrams per day, we calculated the daily consumption of foods (coffee and energy drinks), in grams or milliliters, from multiplying the daily frequency and the size of the portion recorded for each food. The calculation of caffeine intake was obtained from the knowledge of caffeine values in 100 grams or milliliters of each food from the USDA Nutrient Database for Standard Reference^19^. The variable “caffeine intake in mg/day” was categorized into quintiles.

#### Physical inactivity

To evaluate the practice of physical activity, the Self-Administered Physical Activity Checklist (SAPAC)^20^ questionnaire was used, and the results were categorized into “no” (≥ 150 minutes of physical activity/week) and “yes” (< 150 minutes of physical activity/week). The screen exposure time – including television, video game, cell phone, tablet, and computer – was assessed by a questionnaire and categorized into < 2 h, 2–4.9 h, and ≥ 5 h.

#### Overweight

To measure the weight of the adolescents, a high-precision scale coupled to the BOD POD Gold Standard (COSMED)^®^ equipment was used; to measure height, a stadiometer (AlturaExata^®^) was used. Nutritional status was then assessed by Body Mass Index (BMI). For adolescents aged 18 years, the z-score of the BMI curves by age, proposed by the WHO^21^, was used, considering the values > 1 standard deviation (SD) as overweight and the values > 2 SD as obesity. For participants aged 19 years, the WHO classification^22^ proposed for adults was used, considering overweight for BMI of 25–29.9 kg/m^2^ and obesity for BMI ≥ 30 kg/m^2^. The adolescents were categorized as overweight (overweight and obese) or not.

#### Sleep

To assess sleep, two self-administered instruments were used separately, which use subjective measures in scales validated in Brazil: Pittsburgh Sleep Quality Index (PSQI)^23^, and Epworth Sleepiness Scale (ESS)^24^.

The PSQI is a questionnaire with 19 questions regarding sleep quality and disorders in the last month, and evaluates seven sleep components (subjective sleep quality, sleep latency, sleep duration, habitual sleep efficiency, sleep disorders, use of sleeping medications, and daytime dysfunction) with scores ranging from 0 to 3, totaling a maximum of 21 points. Scores greater than 5 indicate poor sleep quality. Other scores were categorized as good sleep quality^23^.

The ESS^24^ is a questionnaire that assesses the probability of napping in eight everyday situations, with the answer to each question ranging from 0 to 3 and with an overall score ranging from 0 to 24. A score ≥ 9 was considered excessive daytime sleepiness. The results were categorized as “normal” or “presence of sleepiness”^14^.

#### Proposed Theoretical Models

Two theoretical models were constructed to estimate the association between the main modifiable risk factors for NCDs and sleep in adolescents, differing only in the variable used to assess the outcome: sleep quality (Model 1) or excessive daytime sleepiness (Model 2). The SES latent variable was the most distal determinant (exogenous variable) associated with all variables of the model. The variables considered behavioral risk factors were the exposures of interest: substance use (tobacco, alcohol, and illicit drugs), unhealthy food consumption (intake of beverages sweetened with sugar and caffeine), screen time, and physical inactivity. Overweight was considered a mediator in the analysis (Figure).

**Figure:**
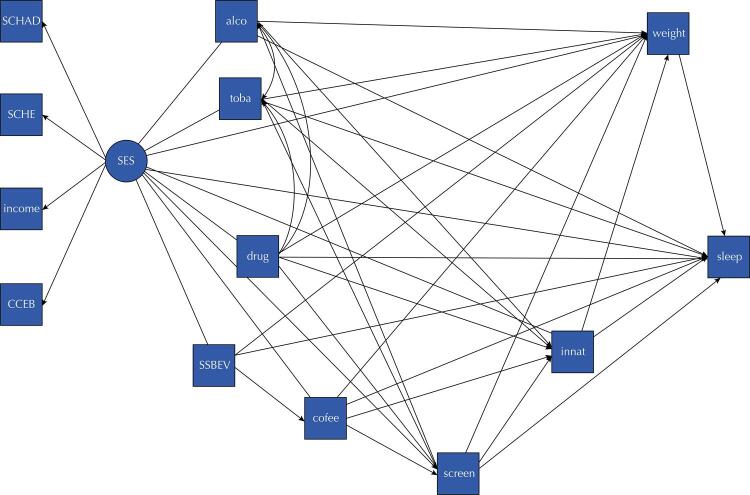
Theoretical model proposed to estimate the associations between behavioral risk factors for NCDs and sleep, mediated by overweight, in adolescents from the 1997–1998 São Luís Birth Cohort. São Luís, MA, Brazil.

Notes: SCHAD: level of education of the adolescent; SCHE: level of education of the head of the family; income: monthly family income; CCEB: Brazilian Economic Classification Criterion; SES: socioeconomic status; alco: alcohol consumption; toba: smoking; drug: use of illicit drugs; SSBEV: consumption of sugar-sweetened beverages; coffee: caffeine consumption; screen: screen time; innat: physical inactivity; weight: overweight; sleep: sleep.

## Statistical Analyses

To investigate the effect of behavioral and metabolic risk factors for NCDs on adolescent sleep, structural equation modeling (SEM) was used. The advantage of this technique is that it allows analyzing the dependency relationships between multiple exposure variables and outcomes, estimating direct and indirect effects, in addition to being able to represent unobserved concepts (latent variables) in these relationships, modeling the measurement error in the estimation process^25^.

Statistical analysis was performed using Mplus software version 7.0. The Weighted Least Squares Mean and Variance Adjusted (WLSMV) estimator was used for continuous and categorical variables and the THETA parameterization was used to control for differences in residual variances. A good latent variable was considered when a factor loading > 0.5 was obtained with a p-value < 0.05. To determine whether the model presented a good fit, the following adjustment indices were considered: value < 0.05 and upper limit of the 90% confidence interval lower than 0.08 for the Root Mean Square Error of Approximation (RMSEA) and values higher than 0.90 for the Comparative Fit Index and the Tucker Lewis Index (CFI/TLI).

The “modindices” command was used to indicate new paths in the initial theoretical model that would result in its best fit. When the proposed modification suggestions were considered plausible from the theoretical point of view, a new model was elaborated and analyzed, if the modification index value was higher than 10. Direct and indirect effects of the observed and latent variables on sleep quality and excessive daytime sleepiness were estimated with standardized coefficients (SC). The effect was considered significant when p < 0.05.

The study met the criteria of Resolution 466/2012 of the National Health Council and Operational Standard 001/2013 CNS; it was approved by the Research Ethics Committee of the University Hospital of UFMA, under the con-substantiated opinion No. 1,302,489 of October 29, 2015.

## RESULTS

In the sample of 2,515 adolescents, 52.5% were female, 69.9% had completed high school, and 14.4% belonged to families in which the head had completed higher education. Regarding economic class, 50.2% of the adolescents were considered class C (Table 1).

**Table 1:** Socioeconomic, behavioral, and sleep characteristics of adolescents from the 1997–1998 São Luís Birth Cohort. São Luís, MA, Brazil.

Characteristic	n	%
Sex		
Male	1,196	47.5
Female	1,319	52.5
Education level of the adolescent		
Primary education	83	3.3
Secondary education	1,758	69.9
Higher education	672	26.8
Education level of the head of the family		
Primary education	593	26.3
Secondary education	1,339	59.3
Higher education	325	14.4
CCEB		
D/E	450	20.2
C	1,116	50.2
B	566	25.4
A	94	4.2
Household monthly income		
≤ 1 MW	802	31.9
< 1–2.9 MW	1,085	43.1
< 3–4.9 MW	341	13.6
≥ 5 MW	287	11.4
Alcohol consumption		
Low risk	2,026	80.6
High risk	489	19.4
Smoking		
No	2,414	96.4
Yes	89	3.6
Use of illicit drugs		
Never used	2,037	81.9
Has used or currently uses	450	18.1
Percentage of energy from sugar-sweetened beverages		
< 5%	535	21.5
5–9.9%	1,093	43.8
≥ 10%	864	34.7
Caffeine intake		
1st quintile	506	20.1
2nd quintile	504	20
3rd quintile	503	20
4th quintile	500	19.9
5th quintile	502	20
Screen time		
< 2 hours	945	37.8
2–4.9 hours	698	27.9
≥ 5 hours	856	34.3
Physical inactivity		
No	1,379	55.1
Yes	1,123	44.9
Overweight		
No	1,905	75.7
Yes	610	24.3
Sleep quality (PSQI)		
Good	979	46.3
Poor	1,137	53.7
Excessive daytime sleepiness (ESS)		
Normal	1,586	63.2
Presence of sleepiness	924	36.8

CCEB: Brazilian Economic Classification Criterion; MW: minimum wage, PSQI: Pittsburgh Sleep Quality Index; ESS: Epworth Sleepiness Scale.

Among the adolescents, 19.4% had high-risk alcohol consumption, 18.1% reported drug use, and 3.6% reported smoking. The consumption of sugar-sweetened beverages was equal to or greater than 10% of the total daily energy intake in 34.7% of the adolescents and caffeine intake had a median of 73.2 mg/day. The percentage of 24.3% of the sample was classified in the overweight range and 44.9% were considered physically inactive.

According to the PSQI, 53.7% of the adolescents had poor sleep quality and, according to the ESS, 36.8% had excessive daytime sleepiness (Table 1).

Both models presented good adjustment according to the RMSEA and CFI indicators (Table 2). The SES latent variable showed evidence of good convergent validity, with all indicator variables presenting factor loading > 0.5 (p < 0.001) (Table 3).

**Table 2:** Adjustment indices of structural equation models for the association between behavioral risk factors for NCDs and sleep, mediated by overweight, in adolescents from the 1997–1998 São Luís Birth Cohort. São Luís, MA, Brazil.

Adjustment rates	Expected values	Observed values	

		Model 1	Model 2
χ^*^			
Value	-	259,706	207,924
Degrees of freedom	-	37	36
p-value	-	< 0.001	< 0.001
RMSEA			
Value	< 0.05	0.049	0.044
90% confidence interval	< 0.08 (upper limit)	0.044–0.055	0.038–0.050

CFI	0.90	0.931	0.947
TLI	0.90	0.854	0.886

Model 1: estimated by the Pittsburgh Sleep Quality Index; Model 2: estimated by the Epworth Sleepiness Scale.

RMSEA: Root Mean Square Error of Approximation; CFI: Comparative Fit Index; TLI: Tucker Lewis Index.

* Chi-square test.

**Table 3:** Factor loading, standard error, and p-value of indicators of the latent variable socioeconomic status of adolescents from the 1997–1998 São Luís Birth Cohort. São Luís, Brazil, 2016.

Latent variable	Model 1			Model 2		
		
Socioeconomic status	Factor loading	Standard error	p-value	Factor loading	Standard error	p-value
Education level of the head of the family	0.665	0.02	< 0.001	0.664	0.02	< 0.001
Education level of the adolescent	0.505	0.024	< 0.001	0.507	0.024	< 0.001
Household monthly income	0.523	0.021	< 0.001	0.524	0.02	< 0.001
Economy class of the family	0.896	0.02	< 0.001	0.895	0.02	< 0.001

Model 1: estimated by the Pittsburgh Sleep Quality Index; Model 2: estimated by the Epworth Sleepiness Scale.

The highest SES showed positive total (SC = 0.128; p < 0.001) and direct (SC = 0.131; p < 0.001) effects on adolescent sleep, when analyzed by means of ESS, which represents greater daytime sleepiness. This effect was not observed when sleep was assessed with the PSQI (SC = 0.026; p = 0.431). High-risk alcohol consumption was associated with worsening in sleep assessed by the ESS (SC = 0.168; p = 0.019) but had only a borderline association by the PSQI (SC = 0.155; p = 0.063). The higher consumption of sugar-sweetened beverages had a negative effect on sleep quality in the two analyzed models (PSQI: SC = 0.089; p = 0.003; ESS: SC = 0.128; p < 0.001) (Table 4).

**Table 4:** Standardized coefficients, standard errors, and p-values of the total, direct, and indirect effects of the explanatory variables on sleep of adolescents from the 1997–1998 São Luís Birth Cohort, tested by Models 1 and 2. São Luís, MA, Brazil.

Explanatory variables	“Sleep”	Total effects			Direct effects			Indirect effects		
		
		SC	SE	p-value	SC	SE	p-value	SC	SE	p-value
SES*	Model 1	0.026	0.033	0.431	0.026	0.034	0.452	0.001	0.01	0.958
	Model 2	0.128	0.03	< 0.001	0.131	0.031	< 0.001	−0.004	0.011	0.747
Alcohol consumption	Model 1	0.155	0.083	0.063	0.176	0.084	0.035	−0.021	0.014	0.137
	Model 2	0.168	0.072	0.019	0.187	0.074	0.012	−0.019	0.014	0.169
Smoking	Model 1	−0.084	0.123	0.497	−0.093	0.122	0.445	0.009	0.018	0.6
	Model 2	−0.129	0.111	0.244	−0.143	0.112	0.201	0.013	0.016	0.401
Use of illicit drugs	Model 1	0.031	0.059	0.604	0.05	0.06	0.405	−0.019	0.011	0.075
	Model 2	0.06	0.057	0.288	0.08	0.059	0.175	−0.019	0.01	0.043
Consumption of sugar-sweetened beverages	Model 1	0.089	0.03	0.003	0.092	0.03	0.002	−0.002	0.002	0.4
	Model 2	0.128	0.024	< 0.001	0.127	0.024	< 0.001	0.002	0.002	0.403
Caffeine intake	Model 1	0.012	0.029	0.667	0.026	0.029	0.367	−0.014	0.006	0.011
	Model 2	0.007	0.027	0.785	0.008	0.028	0.78	0	0.005	0.956
Screen time	Model 1	0.007	0.032	0.821	0.016	0.032	0.607	−0.009	0.005	0.072
	Model 2	−0.05	0.03	0.092	−0.044	0.03	0.145	−0.006	0.004	0.118
Physical inactivity	Model 1	0.147	0.037	< 0.001	0.144	0.037	< 0.001	0.003	0.003	0.287
	Model 2	0.112	0.035	0.001	0.114	0.035	0.001	−0.002	0.003	0.366
Overweight	Model 1	-	-	-	0.061	0.039	0.115	-	-	-
	Model 2	-	-	-	−0.046	0.035	0.195	-	-	-

SC: standardized coefficient; SE: standard error; SES: socioeconomic status.

Model 1: estimated by the Pittsburgh Sleep Quality Index; Model 2: estimated by the Epworth Sleepiness Scale.

* Latent variable defined by the education level of the head of the family, of the adolescent, monthly family income, and economic class of the family.

Physical inactivity was also associated with worse sleep indicators: poor sleep quality (PSQI: SC = 0.147; p < 0.00) and excessive daytime sleepiness (ESS: SC = 0.112; p < 0.001).

No effects of smoking, illicit drug use, caffeine consumption, screen time, and overweight were observed on adolescents’ sleep in the analyzed models (Table 4).

## DISCUSSION

In this study, risk factors for NCDs, such as physical inactivity, increased consumption of alcohol and sugar-sweetened beverages were associated with excessive daytime sleepiness in adolescents. More distally, higher socioeconomic status was also associated with excessive daytime sleepiness in adolescents. Physical inactivity and higher consumption of sugar-sweetened beverages were also associated with poor sleep quality. Overweight was neither a mediator nor associated with sleep quality or excessive daytime sleepiness in our sample.

Physical inactivity was associated with worse sleep parameters analyzed in our study: poor sleep quality and excessive daytime sleepiness. A study with a representative sample of the population of Canada showed that sedentary young men were more likely to have short-term sleep^7^. Physical exercise has been pointed out as an important behavioral treatment to improve sleep quality, in addition to preventing sleep disorders^26^. The beneficial effects of physical activity on sleep can be explained by multiple pathways, for example by the interaction of circadian rhythm and metabolic, vascular, thermoregulatory, immunological, endocrine, and mood effects^26^.

Higher consumption of sugar-sweetened beverages was also associated with worse sleep parameters: poor sleep quality and excessive daytime sleepiness. The consumption of sugar-sweetened beverages has been associated with poor sleep quality^27^, shorter sleep, and later bedtime in adolescents^6^. The consumption of sugar-sweetened beverages can lead to a short duration of sleep, especially when consumed close to bedtime^6^, due to the stimulating properties of sugar and caffeine, which may be present in some of these drinks. The results of this study add knowledge by showing that caffeine consumption was not associated with poor sleep quality or excessive daytime sleepiness in adolescents, which suggests that the association may stem from the effect of sugar.

A higher consumption of sugar-sweetened beverages was also associated with excessive daytime sleepiness, which may be explained by the high-sugar intake resulting in less restorative sleep and more nocturnal awakenings^28^.

The best socioeconomic status was directly associated with excessive daytime sleepiness. These findings are contrary to those of a systematic review, which included 12 articles, showing that lower socioeconomic status was associated with shorter sleep duration, worse subjective perception of sleep quality (assessed with questionnaires), and greater daytime sleepiness in adolescents^9^. However, another study conducted in Brazil, involving adolescents from the city of São Paulo, showed that those with better socioeconomic status had shorter sleep duration^29^. Excessive screen time (≥ 2 hours/day) could be an explanation for this association, since the study of cardiovascular risks in adolescents (ERICA) observed that a better socioeconomic situation was associated with excessive screen time in adolescents from some Brazilian regions, including the Northeast^30^.

Screen time showed no association with sleep among adolescents in this study. Screen time did not even mediate associations in the analysis, even with most adolescents (62.2%) reporting screen time equal to or greater than two hours per day. A systematic review, conducted with 42 articles, aiming to analyze the evidence on the sleep of adolescents, concluded that screen time is an increasingly frequent factor and that it has affected the onset and duration of sleep, with consequent daytime sleepiness, tiredness, and decreased academic performance of adolescents^31^. However, one of the studies in this review, conducted in Hong Kong (n = 762), observed that the time of exposure to television and computer was not associated with sleep duration and quality, and this association was observed only for the use of mobile phones and portable devices to watch videos^32^. In our study, the variable screen time included joint exposure to television, video games, cell phones, tablets, and computers, which may possibly explain the different findings.

Alcohol consumption presented the highest standardized coefficient associated with excessive daytime sleepiness. Alcohol consumption is associated with altered sleep-wake programming and tends to disrupt sleep, especially during the second half of the night’s sleep, exacerbating daytime sleepiness and decreasing alertness. This interruption of sleep continuity in the second half is interpreted as a “rebound effect,” since the alcohol has already been eliminated from the body. This effect happens as the body adjusts to the presence of alcohol during the first half of sleep in an effort to maintain the normal sleep pattern^33^. A U.S. study in two cohorts observed that alcohol use at baseline was negatively associated with weekday sleep and total sleep; and at the two-year follow-up, alcohol consumption was positively associated only with later bedtime in the weekend. The authors highlight that both the pattern and duration of sleep and substance use among young people are interconnected by bidirectional associations^34^; thus, defining causality in this relationship is difficult.

Despite the known effect of being overweight on increased sleep problems^35^ – short-term sleep, more nocturnal awakenings, delayed sleep onset, and sleep-disordered breathing – we found no effect of overweight on sleep quality or daytime sleepiness of adolescents in São Luís. In addition, excess weight did not act as a mediator of associations in the studied models. In this context, note that the city of São Luís is in the Northeast region of Brazil, and this region has lower prevalence of overweight in adolescents when compared with other Brazilian regions, such as the South and Southeast^36^.

This study has some limitations. The use of different scales in the literature, for assessing both sleep and other variables used, such as socioeconomic status, hinders the comparison of results. The study has a cross-sectional design, thus establishing a causal relationship between the risk factors for NCDs and sleep is impossible due to the possibility of reverse causality and the bidirectional relationship between exposures and outcome. However, this is one of the few studies that evaluated the effect of multiple risk factors for NCDs in association with sleep quality and excessive daytime sleepiness in adolescents.

Strengths include the sample size and the method of analysis used to evaluate the effect of the associations. The modeling with structural equations allowed to observe the paths of the effects from mediating variables in sleep, estimating several separate and interdependent multiple regression equations.

## CONCLUSION

The main modifiable behavioral risk factors for NCDs are associated with worse sleep indicators in adolescents, such as poor sleep quality and excessive daytime sleepiness, and are independent of overweight. These findings contribute to emphasize the importance of coordinated surveillance and prevention actions against NCDs and altered sleep, focusing on behavioral risk factors, which begin in adolescence and can trigger health problems throughout life.
