# Supplementary material for: Modifiable behavioral risk factors for NCDs and sleep in Brazilian adolescents
Source: Rev Saude Publica. 2023 Sep 14;57:60. doi: 10.11606/s1518-8787.2023057004957 (PMC10519685; doi:10.11606/s1518-8787.2023057004957)
Supplement: Translation

# Translation: Fatores de risco comportamentais modificáveis para DNT e sono em adolescentes brasileiros

**Authors:** Raina Jansen Cutrim Propp Lima, Mônica Araujo Batalha, Cecília Cláudia Costa Ribeiro, Pedro Martins Lima, Antônio Augusto Moura da Silva, Rosângela Fernandes Lucena Batista

**Affiliations:** I Brasil Instituto Federal de Educação. Ciência e Tecnologia do Maranhão. Departamento de Ensino. Açailândia, MA, Brasil; II Brasil Universidade Federal do Maranhão. Centro de Ciências Biológicas e da Saúde. Departamento de Saúde Pública. São Luís, MA, Brasil; III Brasil Universidade Federal do Maranhão. Centro de Ciências Biológicas e da Saúde. Departamento de Odontologia II. São Luís, MA, Brasil; IV Brasil Universidade Federal do Maranhão. Centro de Ciências Sociais, Saúde e Tecnologia. Imperatriz, MA, Brasil

**Keywords:** Sono, Saúde do Adolescente, Sonolência, Comportamento Sedentário, Consumo de Bebidas Alcoólicas, Bebidas Adoçadas com Açúcar

## Abstract

**OBJETIVO:**

Analisar a associação entre fatores de risco comportamentais modificáveis para doenças não transmissíveis e parâmetros do sono em adolescentes brasileiros.

**MÉTODOS:**

Estudo transversal que utilizou dados do Consórcio de Coortes RPS, São Luís, Brasil para o seguimento de adolescentes de 18-19 anos (n = 2.515). Os desfechos foram a sonolência diurna excessiva (Escala de Sonolência de Epworth – ESE) e a qualidade do sono (Índice de Qualidade do Sono de Pittsburgh – IQSP). As exposições de interesse foram os fatores de risco comportamentais para doenças não transmissíveis (DNT): tempo de tela, inatividade física, álcool, cigarro, drogas ilícitas, consumo de cafeína, consumo de bebidas adoçadas com açúcar. O excesso de peso foi considerado um possível mediador dessa associação entre as exposições de interesse e os desfechos. Os modelos foram analisados por modelagem com equações estruturais.

**RESULTADOS:**

A inatividade física (Coeficiente padronizado, CP = 0,112; p = 0,001), maior consumo de álcool (CP = 0,168; p = 0,019) e de bebidas adoçadas com açúcar (CP = 0,128; p < 0,001) foram associados a sonolência diurna excessiva nos adolescentes; a melhor situação socioeconômica também foi associada a este desfecho (CP = 0,128; p < 0,001). A inatividade física (CP = 0,147; p < 0,001) e o maior consumo de bebidas adoçadas com açúcar (CP = 0,089; p = 0,003) também se associaram com a qualidade do sono ruim. O excesso de peso não foi mediador e nem associado à qualidade do sono ou à sonolência diurna excessiva.

**CONCLUSÕES:**

Os principais fatores de risco comportamentais modificáveis para DNT estão associados a piores parâmetros do sono já na adolescência; alertando para um quadro de acúmulos de riscos para distúrbios de sono no futuro.

## INTRODUÇÃO

O sono é um processo biológico essencial para a sobrevivência, sendo importante para o bem-estar físico, mental e social dos indivíduos^1^. Por ser essencial para o desenvolvimento saudável, a American Academy of Sleep Medicine recomenda que adolescentes devam dormir regularmente de 8 a 10 horas por dia, tempo este associado a melhores desfechos de saúde e qualidade de vida^2^.

A adolescência é um período marcado por mudanças nos padrões de sono devidos a fatores biológicos, ambientais e psicossociais, como maturação puberal, regulação do ritmo circadiano e horários menos regulares^3^. Essas mudanças caracterizam-se por disfunções diurnas do sono, que, por sua vez, são fatores de risco para a sonolência diurna excessiva nessa faixa etária^4^. Reconhece-se que adolescentes não estão dormindo o suficiente, sendo esse um problema crônico em nível mundial. No entanto, muitos dos fatores que contribuem para a atual “epidemia” de sono insuficiente em adolescentes são modificáveis^5^.

Fatores comportamentais, psicossociais e metabólicos do adolescente, como consumo de drogas lícitas e ilícitas, excesso de peso, ingestão elevada de cafeína^3^ e de açúcar adicionado^6^, comportamento sedentário^7^, tempo de tela^8^ e situação socioeconômica^9^ têm sido associados a padrões de qualidade do sono ruim e sonolência diurna excessiva.

A perda do sono e seus distúrbios podem ter seus efeitos acumulados ao longo do tempo, associando-se a diversas consequências prejudiciais à saúde, tais como as doenças não transmissíveis (DNT)^10^. Alguns dos fatores que levam às alterações no sono são considerados de risco para as principais DNT. De acordo com a Organização Mundial de Saúde (OMS)^11^, as DNT estão associadas causalmente a quatro fatores de risco comportamentais modificáveis: uso nocivo de álcool, tabagismo, dieta pouco saudável e inatividade física, que podem desencadear alterações metabólicas como a obesidade.

A epidemia da obesidade é uma realidade em todas as faixas etárias e tem sido considerada uma das principais causas da quantidade de DNT no mundo^12^. O sono de qualidade ruim tem sido associado a maiores taxas de excesso de peso, assim como a obesidade pode desencadear comorbidades que afetam a qualidade do sono. Nessa relação bidirecional^13^, é plausível que fatores de riscos comportamentais influenciem tanto a obesidade quanto o sono; tais fatores serão investigados nesse estudo.

Os riscos comportamentais à saúde mais frequentes na adolescência atuam em conjunto como parte de um estilo não saudável e aumentam o risco da obesidade; por isso, torna-se necessário compreender melhor a associação complexa entre esses fatores de risco e o sono em população adolescente, visto que essa combinação pode impactar a saúde ao longo da vida. Além disso, nenhum estudo que conhecemos utilizou o excesso de peso como mediador dessas associações.

O objetivo deste estudo, portanto, foi estimar, por meio de modelagem com equações estruturais, a associação entre fatores de risco comportamentais modificáveis para DNT e qualidade do sono e sonolência diurna excessiva em adolescentes, considerando o excesso de peso como um mediador desses caminhos.

## MÉTODOS

### Delineamento do Estudo

Estudo transversal aninhado a um estudo de coorte desenvolvido na cidade de São Luís, Maranhão. Essa coorte faz parte da pesquisa intitulada “Determinantes ao longo do ciclo vital da obesidade, precursores de doenças crônicas, capital humano e saúde mental”, desenvolvida pelo Consórcio RPS (Ribeirão Preto, Pelotas e São Luís) de Coortes de Nascimento Brasileiras, que compreende a Faculdade de Medicina de Ribeirão Preto da Universidade de São Paulo (USP), a Universidade Federal de Pelotas (UFPel) e a Universidade Federal do Maranhão (UFMA).

Em São Luís, a coorte de nascimento teve início em 1997 (n = 2.443) e inclui dois seguimentos: o primeiro aos 7-9 anos e o segundo aos 18-19 anos de idade^14^.

No presente estudo foram utilizados dados coletados no segundo seguimento da coorte, obtidos no ano de 2016. Todos os participantes da primeira fase do estudo foram buscados nas quatro juntas de Alistamento Militar na ilha de São Luís, no censo escolar de 2014 e em universidades. Os identificados foram convidados a comparecer ao seguimento, totalizando 687 participantes.

Com o objetivo de aumentar o poder da amostra e para prevenir perdas futuras, a coorte foi aberta para incluir outros indivíduos nascidos no ano de 1997. Em uma primeira etapa esses indivíduos foram incluídos a partir de sorteio, utilizando o banco do Sistema de Informação sobre Nascidos Vivos (SINASC); em uma segunda etapa, incluíram-se voluntários identificados nas escolas e universidades, totalizando 1.828 participantes. A amostra final foi de 2.515 adolescentes.

Os dados foram coletados por alunos e profissionais da área da saúde devidamente treinados, nas dependências da UFMA, que conduziram entrevistas para aplicação de questionários estruturados respondidos pelos próprios participantes. Foram utilizadas as seguintes informações: escolaridade do chefe da família e do adolescente, renda familiar mensal, classe econômica, consumo de álcool, tabagismo, uso de drogas ilícitas, tempo de exposição a telas, consumo alimentar, nível de atividade física, qualidade do sono e sonolência diurna.

### Variáveis Utilizadas

#### Situação socioeconômica

O construto situação socioeconômica (SES) é uma variável latente, derivada a partir das variáveis observadas: nível de escolaridade do chefe da família e do adolescente (ensino fundamental, médio e superior); renda familiar mensal em salários mínimos – em 2016, correspondia a R$ 880,00 (< 1; 1 a 2,9; 3 a 4,9; ≥ 5); e classe econômica familiar, categorizada de acordo com o Critério de Classificação Econômica Brasil (CCEB) 2016^15^, em D/E (classe mais pobre e menos instruída) ou C, B e A (classe mais rica e escolarizada).

#### Consumo de álcool

O padrão de consumo de álcool foi avaliado por meio do instrumento Alcohol Use Disorder Identification Test (AUDIT) e categorizado em baixo risco (0–7) e alto risco (8–40)^16^.

#### Tabagismo

Avaliado por meio da investigação de fumo atual, independente da frequência e/ou quantidade, e dicotomizado em sim ou não.

#### Uso de drogas ilícitas

Uso de drogas ilícitas (maconha, cocaína, heroína, ecstasy, crack, LSD) foi avaliado por meio de questionário autoaplicado e dicotomizado em “nunca usou” e “já usou ou usa atualmente”.

#### Consumo de bebidas adoçadas com açúcar

A frequência de consumo diária de bebidas adoçadas com açúcar e o consumo de cafeína foram obtidos por meio de questionário de frequência alimentar (QFA) validado para adolescentes^17^ e adaptado ao consumo alimentar de adolescentes de São Luís, Maranhão. O questionário continha 106 itens alimentares, que avaliam a frequência e a porção de consumo desses alimentos nos últimos 12 meses. O consumo de bebidas adoçadas com açúcar foi estimado pela ingestão de refrigerantes, sucos industrializados, achocolatados e bebidas energéticas. O percentual de calorias dessas bebidas em relação à ingestão energética diária do adolescente foi calculado pela soma das calorias provenientes de todas as bebidas adoçadas com açúcar, multiplicada por 100 e dividida pelo somatório da ingestão energética diária total. O resultado foi categorizado em < 5%, ≥ 5% e < 10%, e ≥ 10%^18^.

#### Consumo de cafeína

Para estimar o consumo de cafeína em miligramas por dia, calculou-se o consumo diário dos alimentos (café e bebidas energéticas) em gramas ou mililitros, a partir da multiplicação da frequência diária e do tamanho da porção registrada para cada alimento. O cálculo da ingestão de cafeína foi obtido a partir do conhecimento dos valores de cafeína em 100 gramas ou mililitros de cada alimento por meio da USDA Nutrient Database for Standard Reference^19^. A variável “consumo de cafeína em mg/dia” foi categorizada em quintis.

#### Inatividade física

Para avaliar a prática de atividade física utilizou-se o questionário Self-Administered Physical Activity Checklist (SAPAC)^20^ e categorizaram-se os resultados em “não” (tempo ≥ 150 minutos de atividade física/semana) e “sim” (tempo < 150 minutos de atividade física/semana). O tempo de exposição a telas – incluindo televisão, videogame, celular, *tablet* e computador – foi avaliado por meio de questionário e categorizado em < 2 h, 2-4,9 h e ≥ 5 h.

#### Excesso de peso

Para aferição de peso dos adolescente utilizou-se a balança de alta precisão acoplada ao equipamento BOD POD Gold Standard (COSMED^®^); para aferição da altura, utilizou-se o estadiômetro (AlturaExata^®^). O estado nutricional foi, então, avaliado pelo Índice de Massa Corporal (IMC). Para os adolescentes com 18 anos utilizou-se o escore-z das curvas de IMC por idade, proposto pela OMS^21^, considerando-se sobrepeso os valores > 1 desvio-padrão (DP) e obesidade os valores > 2DP. Para os participantes com 19 anos, a classificação da OMS^22^ proposta para adultos foi utilizada, considerando-se sobrepeso para IMC de 25-29,9kg/m^2^ e obesidade IMC ≥ 30kg/m^2^. Os adolescentes foram categorizados como portadores de excesso de peso (sobrepeso e obesidade) ou não.

#### Sono

Para avaliar o sono foram utilizados, separadamente, dois instrumentos autoaplicáveis, que utilizam medidas subjetivas em escalas validadas no Brasil: Índice de Qualidade do Sono de Pittsburgh (IQSP)^23^, e Escala de Sonolência de Epworth (ESE)^24^.

O IQSP é um questionário com 19 perguntas referentes a qualidade e distúrbios do sono no último mês, e avalia sete componentes do sono (qualidade subjetiva do sono, latência para o sono, duração do sono, eficiência habitual do sono, transtornos do sono, uso de medicamentos para dormir e disfunção diurna) por meio de escores que variam de 0 a 3, totalizando um máximo de 21 pontos. Pontuações maiores que 5 indicam qualidade do sono ruim. Outras pontuações foram categorizadas como qualidade do sono boa^23^.

A ESE^24^ é um questionário que avalia a probabilidade de cochilar em oito situações cotidianas, com a resposta de cada questão podendo variar de 0 a 3 e com um escore global variando de 0 a 24. Foi considerada presença de sonolência diurna excessiva escore ≥ 9. Os resultados foram categorizados em “normal” ou “presença de sonolência”^14^.

#### Modelos Teóricos Propostos

Dois modelos teóricos foram construídos para estimar a associação entre os principais fatores de risco modificáveis para DNT e o sono em adolescentes, diferindo somente a variável utilizada para avaliar o desfecho: qualidade do sono (Modelo 1) ou sonolência diurna excessiva (Modelo 2). A variável latente SES foi o determinante mais distal (variável exógena) associado a todas as variáveis do modelo. As variáveis consideradas fatores comportamentais de risco foram as exposições de interesse: uso de substâncias (tabaco, álcool e drogas ilícitas), consumo alimentar não saudável (ingestão de bebidas adoçadas com açúcar e cafeína), tempo de tela e inatividade física. O excesso de peso foi considerado um mediador na análise (Figura).

**Figura:** Modelo teórico proposto para estimar as associações entre os fatores de risco comportamentais para DNT e sono, mediado pelo excesso de peso, em adolescentes da Coorte de Nascimentos de São Luís de 1997–1998.. São Luís, Brasil, 2016.

Notas: ESCAD: nível de escolaridade do adolescente; ESCHE: nível de escolaridade do chefe da família; renda: renda família mensal; CCEB: Critério de Classificação Econômica no Brasil; SES: situação socioeconômica; alco: consumo de álcool; taba: tabagismo; drog: uso de drogas ilícitas; BRAA: consumo de bebidas adoçadas com açúcar; cafe: consumo de cafeína; tela: tempo de tela; inat: inatividade física; peso: excesso de peso; sono: sono.

## Análise Estatística

Para se investigar o efeito dos fatores de risco comportamentais e metabólicos para as DNT sobre o sono de adolescentes foi utilizada a modelagem com equações estruturais (MEE). A vantagem desta técnica é que ela permite analisar as relações de dependência entre múltiplas variáveis de exposição e desfechos, estimando efeitos diretos e indiretos, além de conseguir representar conceitos não observados (variáveis latentes) nessas relações, modelando o erro de mensuração no processo de estimação^25^.

A análise estatística foi realizada usando o software Mplus versão 7.0. Foi utilizado o estimador Weighted Least Squares Mean and Variance Adjusted (WLSMV) para variáveis contínuas e categóricas e a parametrização THETA para controlar diferenças nas variâncias residuais. Foi considerada boa variável latente quando se obteve carga fatorial > 0,5 com p-valor < 0,05. Para determinar se o modelo apresentou um bom ajuste, foram considerados os índices de ajuste: valor < 0,05 e o limite superior do intervalo de confiança de 90% inferior a 0,08 para o Root Mean Square Error of Approximation (RMSEA) e valores superiores a 0,90 para o Comparative Fit Index e o Tucker Lewis Index (CFI/TLI).

O comando “modindices” foi utilizado para indicar novos caminhos no modelo teórico inicial que resultariam em seu melhor ajuste. Quando as sugestões de modificações propostas foram consideradas plausíveis do ponto de vista teórico, um novo modelo foi elaborado e analisado, caso o valor do índice de modificação fosse superior a 10. Foram estimados, por meio de coeficientes padronizados (CP), efeitos diretos e indiretos das variáveis observadas e latentes na qualidade do sono e sonolência diurna excessiva. O efeito foi considerado significativo quando p < 0,05.

O estudo atendeu aos critérios da Resolução 466/2012 do Conselho Nacional de Saúde e da Norma Operacional 001/2013 CNS; foi aprovado pelo Comitê de Ética em Pesquisa do Hospital Universitário da UFMA, sob parecer consubstanciado n° 1.302.489 de 29 de outubro de 2015.

## RESULTADOS

Na amostra de 2.515 adolescentes, 52,5% eram do sexo feminino, 69,9% tinham ensino médio completo e 14,4% pertenciam a famílias em que o chefe tinha ensino superior completo. Em relação à classe econômica, 50,2% dos adolescentes foram considerados da classe C (Tabela 1).

**Tabela 1:** Características socioeconômicas, comportamentais e de sono de adolescentes da Coorte de Nascimentos de São Luís de 1997–1998. São Luís, Brasil, 2016.

Variáveis	n	%
Sexo		
Masculino	1.196	47,5
Feminino	1.319	52,5
Nível de escolaridade do adolescente		
Ensino fundamental	83	3,3
Ensino médio	1.758	69,9
Ensino superior	672	26,8
Nível de escolaridade do chefe da família		
Ensino fundamental	593	26,3
Ensino médio	1.339	59,3
Ensino superior	325	14,4
CCEB		
D/E	450	20,2
C	1.116	50,2
B	566	25,4
A	94	4,2
Renda familiar mensal		
≤ 1 SM	802	31,9
1–2,9 SM	1.085	43,1
3–4,9 SM	341	13,6
≥ 5 SM	287	11,4
Consumo de álcool		
Baixo risco	2.026	80,6
Alto risco	489	19,4
Tabagismo		
Não	2.414	96,4
Sim	89	3,6
Uso de drogas ilícitas		
Nunca usou	2.037	81,9
Já usou ou usa atualmente	450	18,1
Percentual de calorias oriundas das bebidas adoçadas com açúcar		
< 5%	535	21,5
5–9,9%	1.093	43,8
≥ 10%	864	34,7
Consumo de cafeína		
1º quintil	506	20,1
2º quintil	504	20
3º quintil	503	20
4º quintil	500	19,9
5º quintil	502	20
Tempo de tela		
< 2 horas	945	37,8
2–4,9 horas	698	27,9
≥ 5 horas	856	34,3
Inatividade física		
Não	1.379	55,1
Sim	1.123	44,9
Excesso de peso		
Não	1.905	75,7
Sim	610	24,3
Qualidade do sono (IQSP)		
Boa	979	46,3
Ruim	1.137	53,7
Sonolência diurna excessiva (ESE)		
Normal	1.586	63,2
Presença de sonolência	924	36,8

CCEB: Critério de Classificação Econômica Brasil; SM: Salário mínimo; IQSP: Índice de Qualidade do Sono de Pittsburgh; ESE: Escala de Sonolência de Epworth.

Entre os adolescentes, 19,4% apresentavam consumo de álcool de alto risco, 18,1% relataram uso de drogas e 3,6%, de cigarros. O consumo de bebidas adoçadas com açúcar foi igual ou maior que 10% da ingestão calórica total diária em 34,7% dos adolescentes e de cafeína teve mediana de 73,2 mg/dia. O percentual de 24,3% da amostra foi classificado na faixa do excesso de peso e 44,9% foram considerados fisicamente inativos.

Segundo o IQSP, 53,7% dos adolescentes tinham qualidade do sono ruim e, segundo a ESE, 36,8% apresentaram sonolência diurna excessiva (Tabela 1).

Os dois modelos apresentaram bom ajuste segundo os indicadores RMSEA e CFI (Tabela 2). A variável latente SES apresentou evidências de boa validade convergente, com todas as variáveis indicadoras apresentando carga fatorial > 0,5 (p < 0,001) (Tabela 3).

**Tabela 2:** Índices de ajuste dos modelos de equações estruturais para a associação entre os fatores de risco comportamentais para DNT e sono, mediado pelo excesso de peso, em adolescentes da Coorte de Nascimentos de São Luís de 1997–1998. São Luís, Brasil, 2016.

Índices de ajuste	Valores esperados	Valores observados	

		Modelo 1	Modelo 2
χ^*^			
Valor	-	259.706	207.924
Graus de liberdade	-	37	36
p-valor	-	< 0,001	< 0,001
RMSEA			
Valor	< 0,05	0,049	0,044
Intervalo de confiança de 90%	< 0,08 (limite superior)	0,044–0,055	0,038–0,050

CFI	0,90	0,931	0,947
TLI	0,90	0,854	0,886

Modelo 1: estimado pelo Índice de Qualidade do Sono de Pittsburgh; Modelo 2: estimado pela Escala de Sonolência de Epworth.

RMSEA: *Root Mean Square Error of Approximation*; CFI: *Comparative Fit Index*; TLI: *Tucker Lewis Index*.

*Teste qui-quadrado.

**Tabela 3:** Carga fatorial, erro padrão e p-valor de indicadores da variável latente situação socioeconômica dos adolescentes da Coorte de Nascimentos de São Luís de 1997–1998. São Luís, Brasil, 2016.

Variável latente	Modelo 1			Modelo 2		
		
Situação socioeconômica	Carga fatorial	Erro padrão	p-valor	Carga fatorial	Erro padrão	p-valor
Escolaridade do chefe da família	0,665	0,02	< 0,001	0,664	0,02	< 0,001
Escolaridade do adolescente	0,505	0,024	< 0,001	0,507	0,024	< 0,001
Rendar familiar mensal	0,523	0,021	< 0,001	0,524	0,02	< 0,001
Classe econômica familiar	0,896	0,02	< 0,001	0,895	0,02	< 0,001

Modelo 1: estimado pelo Índice de Qualidade do Sono de Pittsburgh; Modelo 2: estimado pela Escala de Sonolência de Epworth.

A maior SES apresentou efeitos total (CP = 0,128; p < 0,001) e direto positivos (CP = 0,131; p < 0,001) no sono dos adolescentes, quando analisado por meio da ESE, o que representa maior sonolência diurna. Tal efeito não foi observado quando o sono foi avaliado pelo IQSP (CP = 0,026; p = 0,431). Consumo de álcool de alto risco foi associado à piora no sono avaliado pela ESE (CP = 0,168; p = 0,019), mas teve somente associação limítrofe pelo IQSP (CP = 0,155; p = 0,063). O maior consumo de bebidas adoçadas com açúcar teve efeito negativo sobre a qualidade do sono nos dois modelos analisados (IQSP: CP = 0,089; p = 0,003; ESE: CP = 0,128; p < 0,001) (Tabela 4).

**Tabela 4:** Coeficientes padronizados, erros padrão e p-valores dos efeitos totais, diretos e indiretos das variáveis explanatórias no sono de adolescentes da Coorte de Nascimentos de São Luís de 1997–1998, testados pelos modelos 1 e 2. São Luís, Brasil, 2016.

Variáveis explanatórias	“Sono”	Efeitos totais			Efeitos diretos			Efeitos indiretos		
		
		CP	EP	p-valor	CP	EP	p-valor	CP	EP	p-valor
SES*	Modelo 1	0,026	0,033	0,431	0,026	0,034	0,452	0,001	0,01	0,958
	Modelo 2	0,128	0,03	< 0,001	0,131	0,031	< 0,001	-0,004	0,011	0,747
Consumo de álcool	Modelo 1	0,155	0,083	0,063	0,176	0,084	0,035	-0,021	0,014	0,137
	Modelo 2	0,168	0,072	0,019	0,187	0,074	0,012	-0,019	0,014	0,169
Tabagismo	Modelo 1	-0,084	0,123	0,497	-0,093	0,122	0,445	0,009	0,018	0,6
	Modelo 2	-0,129	0,111	0,244	-0,143	0,112	0,201	0,013	0,016	0,401
Uso de drogas ilícitas	Modelo 1	0,031	0,059	0,604	0,05	0,06	0,405	-0,019	0,011	0,075
	Modelo 2	0,06	0,057	0,288	0,08	0,059	0,175	-0,019	0,01	0,043
Consumo de bebidas adoçadas com açúcar	Modelo 1	0,089	0,03	0,003	0,092	0,03	0,002	-0,002	0,002	0,4
	Modelo 2	0,128	0,024	< 0,001	0,127	0,024	< 0,001	0,002	0,002	0,403
Consumo de cafeína	Modelo 1	0,012	0,029	0,667	0,026	0,029	0,367	-0,014	0,006	0,011
	Modelo 2	0,007	0,027	0,785	0,008	0,028	0,78	0	0,005	0,956
Tempo de tela	Modelo 1	0,007	0,032	0,821	0,016	0,032	0,607	-0,009	0,005	0,072
	Modelo 2	-0,05	0,03	0,092	-0,044	0,03	0,145	-0,006	0,004	0,118
Inatividade física	Modelo 1	0,147	0,037	< 0,001	0,144	0,037	< 0,001	0,003	0,003	0,287
	Modelo 2	0,112	0,035	0,001	0,114	0,035	0,001	-0,002	0,003	0,366
Excesso de peso	Modelo 1	-	-	-	0,061	0,039	0,115	-	-	-
	Modelo 2	-	-	-	-0,046	0,035	0,195	-	-	-

CP: coeficiente padronizado; EP: Erro Padrão; SES: situação socieconômica.

Modelo 1: estimado pelo Índice de Qualidade do Sono de Pittsburgh; Modelo 2: estimado pela Escala de Sonolência de Epworth.

* Variável latente definida por escolaridade do chefe da família, escolaridade do adolescente, renda familiar mensal e classe econômica familiar.

A inatividade física também foi associada a piores indicadores do sono: qualidade ruim do sono (IQSP: CP = 0,147; p < 0,00) e sonolência diurna excessiva (ESE: CP = 0,112; p < 0,001).

Não foram observados efeitos do tabagismo, uso de drogas ilícitas, consumo de cafeína, tempo de tela e excesso de peso no sono dos adolescentes nos modelos analisados (Tabela 4).

## DISCUSSÃO

Neste estudo, os fatores de risco para DNT como inatividade física, maior consumo de álcool e de bebidas adoçadas com açúcar foram associados à sonolência diurna excessiva nos adolescentes. Mais distalmente, a maior situação socioeconômica também se associou à sonolência diurna excessiva nos adolescentes. A inatividade física e o maior consumo de bebidas adoçadas com açúcar se associaram também à qualidade do sono ruim. Em nossa amostra, o excesso de peso não foi mediador e nem esteve associado à qualidade do sono e sonolência diurna excessiva.

A inatividade física foi associada a piores parâmetros do sono analisados em nosso estudo: qualidade ruim do sono e sonolência diurna excessiva. Estudo com amostra representativa da população do Canadá mostrou que jovens sedentários do sexo masculino tiveram maior probabilidade de ter sono de curta duração^7^. O exercício físico tem sido apontado como um tratamento comportamental importante para melhorar a qualidade do sono, além de prevenir distúrbios do sono^26^. Os efeitos benéficos da prática de atividade física sobre o sono podem ser explicados por múltiplos caminhos, por exemplo pela interação do ritmo circadiano, efeitos metabólicos, vasculares, termorregulatórios, imunológicos, endócrinos e do humor^26^.

O maior consumo de bebidas adoçadas com açúcar também se associou a piores parâmetros do sono: qualidade do sono ruim e sonolência diurna excessiva. A ingestão de bebidas adoçadas com açúcar tem sido associada à qualidade do sono ruim^27^, sono mais curto e horário de dormir mais tardio em adolescentes^6^. A ingestão de bebidas adoçadas com açúcar pode levar a uma curta duração do sono, principalmente quando consumidas próximo à hora de dormir^6^, devido às propriedades estimulantes do açúcar e da cafeína, que pode estar presente em algumas dessas bebidas. Os resultados do presente estudo adicionam conhecimento ao mostrar que o consumo de cafeína não foi associado à qualidade do sono ruim nem à sonolência diurna excessiva nos adolescentes, o que sugere que a associação possa decorrer do efeito do açúcar.

Um maior consumo de bebidas adoçadas com açúcar também esteve associado à sonolência diurna excessiva, o que pode ser explicado por a ingestão de alto teor de açúcar resultar num sono menos restaurador e mais despertares noturnos^28^.

A melhor situação socioeconômica foi diretamente associada à sonolência diurna excessiva. Estes achados são contrários ao de uma revisão sistemática, que incluiu 12 artigos, mostrando que menor *status* socioeconômico se associou à menor duração do sono, à pior percepção subjetiva da qualidade do sono (avaliada por meio de questionários), à maior sonolência diurna em adolescentes^9^. Entretanto, outro estudo conduzido no Brasil, envolvendo adolescentes da cidade de São Paulo, mostrou que aqueles com melhor *status* socioeconômico tiveram menor duração do sono^29^. O tempo excessivo de tela (≥ 2 horas/dia) poderia ser uma explicação para essa associação, pois no estudo de riscos cardiovasculares em adolescentes (ERICA) foi observado que a melhor situação socioeconômica foi associada ao tempo excessivo de tela nos adolescentes de algumas regiões brasileiras, incluindo o Nordeste^30^.

Não foi encontrada associação entre o tempo de tela e sono dos adolescentes no presente estudo. Tempo de tela sequer mediou associações na análise, mesmo com grande parte dos adolescentes (62,2%) relatando tempo de tela igual ou superior a duas horas diárias. Revisão sistemática, realizada com 42 artigos, com objetivo de analisar as evidências sobre o sono dos adolescentes, concluiu que o tempo de tela é um fator cada vez mais frequente e que tem afetado o início e a duração do sono, com consequente sonolência diurna, cansaço e diminuição do desempenho acadêmico de adolescentes^31^. Entretanto, um dos estudos dessa revisão, realizado em Hong Kong (n = 762), observou que o tempo de exposição à televisão e ao computador não foi associado à duração e qualidade do sono, sendo essa associação observada apenas para o uso de celular e dispositivos portáteis para assistir vídeos^32^. No presente estudo, a variável tempo de tela incluiu a exposição conjunta à televisão, ao videogame, ao celular, ao tablet e ao computador o que pode ser uma possível explicação para os diferentes achados.

Consumo de álcool apresentou o mais alto coeficiente padronizado na associação com a sonolência diurna excessiva. O consumo de álcool está associado à programação de sono-vigília alterada e tende a perturbar o sono, especialmente durante a segunda metade da noite de sono, exacerbando a sonolência diurna e diminuindo o estado de alerta. Essa interrupção da continuidade do sono na segunda metade é interpretada como um “efeito rebote”, já que o álcool já foi eliminado do corpo. Este efeito acontece como ajuste do corpo à presença de álcool durante a primeira metade do sono em esforço para manter o padrão normal de sono^33^. Um estudo norte-americano em duas coortes, observou que o uso de álcool no *baseline* foi associado negativamente ao sono durante a semana e ao sono total; e no seguimento dos dois anos, o consumo de álcool esteve associado positivamente somente à hora de dormir mais tarde no final de semana. Os autores destacam que tanto o padrão quanto a duração do sono e o uso de substâncias entre os jovens estão interligados por meio de associações bidirecionais^34^, sendo difícil definir a causalidade nessa relação.

Apesar do efeito conhecido do excesso de peso no aumento de problemas do sono^35^ – sono de curta duração, mais despertares noturnos, atraso no início do sono e distúrbios respiratórios do sono – não encontramos nenhum efeito do excesso de peso na qualidade do sono ou na sonolência diurna dos adolescentes em São Luís. Além disso, o excesso de peso não atuou como mediador de associações nos modelos estudados. Neste contexto, é importante destacar que a cidade de São Luís, encontra-se na região Nordeste do Brasil e essa região apresenta menores prevalências de excesso de peso em adolescentes quando comparada a outras regiões brasileiras, como Sul e Sudeste^36^.

Este estudo tem limitações. A utilização de diferentes escalas na literatura, no que se refere à avaliação tanto do sono quanto de outras variáveis utilizadas, como situação socioeconômica, dificulta a comparação entre resultados. O estudo tem desenho transversal, dessa forma, não é possível estabelecer uma relação causal entre os fatores de risco para DNT e o sono, por conta da possibilidade de causalidade reversa e da relação bidirecional existente entre exposições e desfecho. Porém, este é um dos poucos estudos que avaliou o efeito de múltiplos fatores de risco para DNT em associação com a qualidade do sono e a sonolência diurna excessiva de adolescentes.

Como pontos fortes temos o tamanho da amostra e o método de análise utilizado para avaliar o efeito das associações. A modelagem com equações estruturais permitiu observar os caminhos dos efeitos através de variáveis mediadores no sono, estimando várias equações de regressão múltipla separadas e interdependentes.

## CONCLUSÃO

Os principais fatores de risco comportamentais modificáveis para DNT estão associados a piores indicadores do sono em adolescentes, como qualidade do sono ruim e sonolência diurna excessiva, e independem do excesso de peso. Esses achados contribuem para enfatizar a importância de ações coordenadas de vigilância e prevenção às DNT e sono alterado, dando maior atenção aos fatores de risco comportamentais, que têm início na adolescência e podem desencadear problemas de saúde ao longo da vida.
